# Tobacco endgame policies: an analysis of preferred strategies and support levels in a sample from Qatar

**DOI:** 10.3389/fpubh.2025.1515633

**Published:** 2025-06-09

**Authors:** Aisha Al-Naimi, Khadiga Elsayed, Marwa Alharoon, Fatma Al-Obaidli, Hissa Almuraikhi, Amaal Osman, Reem Al-Rashdi, Mujahed Shraim, Mohammed Al-Hamdani

**Affiliations:** Department of Public Health, College of Health Sciences, QU Health, Qatar University, Doha, Qatar

**Keywords:** tobacco endgame, tobacco control, policy support, tobacco use status, policy preferences, Qatar, sex differences

## Abstract

**Background:**

There is limited evidence on preferences for tobacco endgame policies and support levels for them in the Middle East. Further, no studies on the interactive association of sex and tobacco use status with support levels exist to date.

**Objectives:**

To examine preferred tobacco endgame strategies and levels of support in a Qatari sample.

**Methods:**

A convenience sample of adults (*N* = 372; 73.3% females) completed a cross sectional survey. Preferences for strategies were assessed by demographic variables using chi-square tests and levels of support were compared by sex and tobacco use status while adjusting for other factors using bootstrapped regression.

**Results:**

Males and current tobacco users prefer standardized packages, females prefer nicotine reduction policies, and never tobacco users prefer tax increases, import bans, bans for minors and adults, and flavor bans. Never and past tobacco users reported higher tobacco endgame support relative to current tobacco users. Further, males who never used tobacco or used it in the past reported higher tobacco endgame support than male current users.

**Conclusion:**

High tobacco endgame support level and preferences for a wide range of tobacco endgame policies might be promising indicators for embracing them, especially among never tobacco users and males that do not currently use tobacco in Qatar.

## Introduction

The tobacco endgame refers to achieving a tobacco-free future or an endpoint with negligible tobacco use levels (1, 2). Endgame strategies represent a deviation from traditional tobacco control policies that slowly reduce tobacco use. They present either a novel strategy such as a sinking lid, which refers to bid quotas to sell in countries with no tobacco imports or an aggressive form of known policies such as very high tax increases (3, 4). They also tend to be clear with measurable goals (5, 6). Tobacco endgame strategies are receiving attention in some countries (7). A number of countries have tobacco endgame goals. For instance, Hong Kong has set a target to achieve this goal by 2022, while Ireland and New Zealand have set their targets for 2025 (7). Scotland has set a more distant goal of 2034, and Wales aims to reach this milestone by 2040 (7). These targets reflect the growing recognition among countries of the urgent need to tackle tobacco use and its adverse health effects, which provide evidence to promote public and political support.

Tobacco endgame strategies are diverse, some have been implemented in select countries and others remaining under consideration (8). Aggressive tobacco tax increases constitute a tobacco endgame strategy—for example: a tax increase of 25% in Australia followed by multiple 12.5% increases is associated with success in terms of prevalence reductions (4, 9). Healthcare cost recovery is another tobacco endgame strategy, which is holding the tobacco industry accountable for healthcare costs incurred due to tobacco use (10). It has been implemented in the United States with notable positive outcomes including the exposure of internal industry documents that were used to propel policy change (11). A third example of a tobacco endgame strategy is plain/standardized packaging which refers to standardizing tobacco product packaging such that the brand imagery is removed, and the font, background color and shape of the package is standardized (12). This type of packaging has been shown to reduce product appeal (13) and increase quit attempts (14) and has been implemented in many countries including Australia, New Zealand, Canada and France (15). A fourth example are age-based restrictions such as generational bans that do not allow individuals born after a certain year from smoking when they become adults (16) or increasing minimum age for tobacco use to 21 (17), the latter is associated with reductions in tobacco use (18).

A fifth example is flavored bans. Brown et al. (19) reported the impact of bans on menthol bans in Ontario, Canada—after 7 months of implementing the ban, there was decrease in sales by 93%, with minimal replacement by non-menthol cigarettes. Similarly, restrictions of flavored tobacco sales was associated with a reduction in the total sales of tobacco products by 25% in San Francisco with no substitution concerns (20).

In a study that modeled the effects of endgame strategies in Ontario Canada, taxes contributed the largest independent effect on smoking prevalence, followed by decreased availability of tobacco (21). van der Deen et al. (8), examined a forecasting smoking prevalence model and a closed cohort model for the population for health benefits and costs that includes 16 tobacco-related diseases to forecast the effect of four endgame strategies. One of these endgame strategies was an ongoing annual tax of 10% for which the prevalence of tobacco use decreased to 34% since 2011 and expected to reach an estimate of 16% in 2025 and a sinking lid on tobacco supply is estimated to result in a tobacco prevalence of 0% by the year 2025 (8). Similarly, a study conducted in Singapore for tobacco endgame strategies found that annual taxes, free smoking cessation programs, banning flavors, and increasing the minimum legal age are the most effective modeled strategies for reducing tobacco prevalence by 2070 (22).

Studies on public opinion about the tobacco endgame signals support for some strategies. For instance, a qualitative study of daily smoking adults in New Zealand revealed that they favor nicotine reduction policies yet oppose finance-based policies, both as an incentive to quit and a disincentive as taxes (23). Another qualitative study of current smokers and past smokers with a recent history belonging to four priority populations: young adults, Maori, Pacific, and pregnant women revealed support for a 2025 smoke-free goal yet asserted the importance of maintaining “their freedom” to smoke (24). Freedom is often believed to be an industry tactic to perpetuate smoking and the authors highlight a need to de-normalization strategies to counter industry messaging (24).

There are differences in public support for endgame strategies by country of residence, tobacco endgame status and other demographic variables. Gallus et al. (25), studied public support for total tobacco bans (an endgame strategy) in a sample of European countries and found that support ranged from 18 to 60%. Differences in support were also different by tobacco use history—never smokers supported total tobacco bans the most followed by past smokers and smokers (25). These differences in support for total bans by tobacco use status were mirrored in Hong Kong (26). In a Danish study, future bans on smoking and tax increases were supported by 30.6 and 59% of the studied participants respectively, with never smokers exhibiting higher likelihood for supporting the measures relative to daily smokers (27). Kang et al. (28), revealed that the tobacco endgame policies with the highest support level in Korea are nicotine reduction policies and retailer restriction policies, and (29), showed that phasing out strategies top the list, especially when coupled with support for addicted users. Kim et al. (30), summarized the evidence on tobacco endgame support in 47 studies from more than nine countries and found a clearly lower level of support by smokers in comparison to non-smokers. Kim et al. (30), also found that the highest level of support for an endgame strategy belonged to very low nicotine level policies.

The decision to support tobacco endgame policies or prefer specific policies can be explained through the Health Belief Model (HBM) (31). In the HBM, the perceived benefits of supporting endgame policies or preferring a specific tobacco endgame policy is based on the perceived susceptibility to tobacco related illnesses from second and third hand smoke for non-tobacco users and first, second and third hand smoke for tobacco users. The perceived severity of illnesses that stem from the exposure to the aforementioned also plays a role in the decision to support or prefer tobacco endgame policies. A number of modifying variables including sex and tobacco use status play a role in influencing a person’s decision to support or prefer the tobacco endgame policies. For instance, past research showed more support for such policies by females versus males, and non-users versus users (24, 25, 32). Further, policy support differ across countries (33).

### Literature gaps and current aim

Despite the evidence on tobacco endgame strategies, studies often examine one or a few types of tobacco endgame strategies, rather than a diverse list of strategies, which limits our understanding on how the support differs across the strategies. For example, total ban support was the only studied strategies in some studies (25, 26). Further, although studies were conducted in in Europe and some countries such as Hong Kong, Denmark, South Korea and Ireland (25–29), none to our knowledge exists in the Middle East.

Research hitherto did not compare support levels by sex and tobacco use status as an interaction. Sex comparisons with respect to tobacco endgame strategies are not often studied, and when done, lack an examination of an interactive effect of sex and tobacco use status, which is crucial given that tobacco use status has been consistently associated with tobacco endgame strategy support [e.g., (25, 26)]. Males tend to have lower support levels for tobacco control policies relative to females (32), yet their preferences for tobacco endgame measures differ—women prefer taxes more and future bans less relative to males (27). Selvan et al. (34), conducted a scoping review of countries with the highest readiness for tobacco endgame policies, and Qatar was listed as one of these countries, yet no studies have examined public support for such policies in the country. Current tobacco use prevalence in the country remains high—25.2% in Qatar relative to 22.3% worldwide (35, 36), thereby warranting an understanding of policy preferences and implement those with high preference levels while raising awareness for policies that are associated with low preference levels. This project will cover the gaps in studying tobacco endgame strategies by including a Middle Eastern sample from Qatar, examining the most preferred tobacco endgame strategies by different demographic variables and comparing the support levels by tobacco use status and sex. Precisely, the study will answer the following research questions: Q1) Do tobacco endgame policy preferences and support levels differ depending on sociodemographic variables? Q2) Do tobacco endgame policy preferences and support levels differ by sex and tobacco use status?

## Materials and methods

### Sample

Both online and offline recruitment was conducted among adults aged 18–60 years at Qatar University. For the offline distribution, flyers were distributed in each campus to students and staff. Online distributions were via email invitations to university staff and students to take part in the survey. The survey, available in two languages, English and Arabic, allowed participants to choose their preferred language. Participants were categorized into groups based on their sex (males vs. females) and tobacco use status (tobacco users, ex-tobacco users, and non-tobacco users). Additionally, an email list of participants who had consented to future studies from a previous research project on tobacco control was utilized to enhance recruitment efficiency. Ethics approval was obtained from the Qatar University IRB (#QU-IRB 1894-E/23).

### Procedure

The research included an online survey using secure survey software. The target population adults at Qatar University. After accessing the link to the survey, an informed consent form appeared. If participants agreed to partake in the study, they provided their consent by clicking on “I agree to participate.” The study consisted of a demographic questionnaire about sex, socioeconomic status, employment, educational level, and tobacco use status. Participants were then asked to choose their most preferred tobacco endgame strategy from a list of 10 tobacco endgame strategies. They were then asked to indicate their level of support for each one of the tobacco endgame strategies based on a Likert scale: “1 strongly oppose, 2 oppose, 3 indifferent, 4 support, or 5 strongly support.” The results of the study are discussed from the perspective of the HBM.

### Sample size calculation

Gpower sample size calculations for the most demanding analysis in this study require a maximum *N* = 359 responses, based on an F test for 4 groups (male vs. female, Tobacco user vs. non-tobacco user), effect size *f* = 0.2 (small to medium effect size), a power of 0.99, Df = 4 = 3 (*N*-1, 4–1) at an alpha level of 0.05. To accomplish this, we aimed to gather data from 1,000 participants to be as close as possible to 359 complete responses.

### Design and analysis

The study’s design is a cross-sectional survey. For the statistical analyses, we used SPSS for chi-square tests to examine the association between sociodemographic variables and preference for tobacco endgame strategy choice. Pairwise differences in preferences for tobacco endgame strategies for each layer of demographic variables were assessed using Bonferroni Correction Tests at *p* < 0.05.

Tobacco endgame support was assessed through a Tobacco Endgame Scale. To develop the scale performed a literature review of endgame policies and noted the identified policies. The comprehensibility of the items was checked through pilot testing. Factor analysis with a Promax rotation was run on the 10-item Tobacco Endgame Scale. Items with less than a loading of 0.33 were considered for removal and those loading at 0.33 or more on two factors were considered cross loaded items. Exploratory factor analysis was used given that the scale was being tested for the first time.

Regression tests were conducted using STATA. The mean of the Tobacco Endgame Scale was computed. It was not normally distributed (skewed to the right). Therefore, linear regression analyses with bootstrap sampling (3,000 bootstrap replications and a random seed number of 12,345) were used to estimate regression coefficients, bootstrap standard errors (SE), and bias-corrected (BC) bootstrap 95% confidence intervals (CI) for the crude and adjusted associations between predictor variables and tobacco endgame scores. Only variables associated with the tobacco endgame scores with a *p*-value ≤ 0.20 in crude associations were included in the multivariable (adjusted) analysis.

## Results

### Summary of sample characteristics

Table 1 represents the sociodemographic of the study sample (*N* = 372). The mean age of the participants was 24.88 (SD: 8.0). In this sample, 73.3% of the participants were females. Only 24.7% of the participants were employed. In addition, 11% of the participants self-described themselves as having high socioeconomic status, 81.5% as medium socioeconomic status, and 7.5% as low socioeconomic status. The most common attained educational level was high school education (46.2%), followed by a Bachelor’s degree (33.3%), and a master’s degree (9.9%). Further, 9.9% were current smokers, 8.1% were past smokers, and 82% of the participants never smoked. Among smokers, 41.9% smoked less than one cigarette per day while 29% smoked 1–10 cigarettes per day. Moreover, the top three preferred tobacco endgame strategies were banning tobacco use for future generations (18%), followed by reducing nicotine to non-addictive levels (16%) and banning imports of tobacco by 10% for ten 10 years to eliminate tobacco availability (15%).

**Table 1:** Sample characteristics.

Variables		n	%	M	SD
Age				24.88	8
Sex	Female	273	73.3		
	Male	99	26.6		
Employed	Yes	92	24.7		
	No	280	75.3		
Socioeconomic status	Low	28	7.5		
	Medium	303	81.5		
	High	41	11		
Education level	Less than High school	1	0.3		
	High school	172	46.2		
	Post-secondary diploma	22	5.9		
	Bachelor’s degree	124	33.3		
	Master’s degree	37	9.9		
	MD degree	1	0.3		
	PhD degree	15	4		
Tobacco use status	Current user	37	9.9		
	Past user	30	8.1		
	Never user	305	82		
How much do you smoke	>1 cigarette per day	13	41.9		
	1–10 cigarettes per day	9	29		
	11–20 cigarettes per day	6	19.4		
	<20 cigarettes per day	3	9.7		
Policies	Ban tobacco use for future generations	67	18		
	Tax increases	52	14		
	Standardizing packages	12	3.2		
	Healthcare cost recovery lawsuits	37	9.9		
	Ban imports	57	15.3		
	Reduce nicotine concentration	60	16.1		
	Ban tobacco use for minors and adults	45	12.1		
	Set the minimum age to 21	9	2.4		
	Set the minimum age to 25	16	4.3		
	Ban all tobacco flavors	17	4.6		

n, number; M, Mean; SD, standard deviation.

### Association between sociodemographic and preferred tobacco endgame strategy

Table 2 displays the associations between sociodemographic variables and preferred tobacco endgame policies. As shown in Table 2, sex was significantly associated with preference for tobacco endgame strategies. For males, there was an overrepresentation of preference for standardized tobacco packaging, in comparison to nicotine reduction policies. For females, there was a significant overrepresentation of preference for nicotine reduction policies in comparison to standardized tobacco packaging. Tobacco use status was also significantly associated with preference for tobacco endgame strategies. For current tobacco users, there was a significant overrepresentation of preference for standardized tobacco packaging in comparison to bans for future generations, tax increases, import bans, nicotine reduction, and full bans for adults and minors. For never tobacco users, there was a significant overrepresentation of preference for tax increases, import bans, bans for minors and adults, and flavor bans relative to standardizing packages. Employment, socioeconomic status and education level were not associated with preference for tobacco endgame strategies.

**Table 2:** Associations between sociodemographic variables and preferred tobacco endgame policy.

				Preferred tobacco endgame policy									
Sociodemographic variables	df	χ^2^	*p*	Ban use for future generations N (%), [adj residual]	Tax increases N (%), [adj residual]	Standardizing packages N (%), [adj residual]	Healthcare cost recovery lawsuits N (%), [adj residual]	Ban imports N (%), [adj residual]	Reduce nicotine concentration N (%), [adj residual]	Ban tobacco use for minors and adults N (%), [adj residual]	Set the minimum age to 21 N (%), [adj residual]	Set minimum age to 25 N (%), [adj residual]	Ban all tobacco flavors N (%), [adj residual]
Employment	9	13.168	0.155										
Yes				11a (16.4) [−1.7]	9a (17.3) [−1.3]	4a (33.3) [0.7]	11a (29.7) [0.7]	14a (24.6) [0.0]	21a (35.0) [2.0]	15a (33.3) [1.4]	2a (22.2) [−0.2]	1a (6.3) [−1.8]	4a (23.5) [−0.1]
No				56a (83.6) [1.7]	43a (82.7) [1.3]	8a (66.7) [−0.7]	26a (70.3) [−0.7]	43a (75.4) [0.0]	39a (65.0) [−2.0]	30a (66.7) [−1.4]	7a (77.8) [0.2]	15a (93.8) [1.8]	13a (76.5) [0.1]
Sex	9	21.08	0.012										
Male				25a,b (37.3) [2.2]	18a,b (34.6) [1.4]	7b (58.3) [2.5]	9a, b (24.3) [−0.3]	15a,b (26.3) [−0.1]	8a (13.3) [−2.5]	11a, b (24.4) [−0.4]	2a,b (22.2) [−0.3]	2a, b (12.5) [−1.3]	2a, b (11.8) [−1.4]
Female				42a,b (62.7) [−2.2]	34a,b (65.4) [−1.4]	5b (41.7) [−2.5]	28a, b (75.7) [0.3]	42a, b (73.7) [0.1]	52a (86.7) [2.5]	34 a, b (75.6) [0.4]	7a,b (77.8) [0.3]	14a, b (87.5) [1.3]	15a, b (88.2) [1.4]
Socioeconomic status	18	24.696	0.134										
Low				6 a (9.0) [0.5]	2a (3.8) [−1.1]	3a (25.0) [2.3]	4a (10.8) [0.8]	5a (8.8) [0.4]	4a (6.7) [−0.3]	1a (2.2) [−1.4]	0a (0.0) [−0.9]	0a (0.0) [−1.2]	3a (17.6) [1.6]
Medium				53a (79.1) [−0.5]	46a (88.5) [1.4]	7a (58.3) [−2.1]	30a (81.1) [−0.1]	51a (89.5) [1.7]	45a (75.0) [−1.4]	38a (84.4) [0.6]	7a (77.8) [−0.3]	14a (87.5) [0.6]	12a (70.6) [−1.2]
High				8a (11.9) [0.3]	4a (7.7) [−0.8]	2a (16.7) [0.6]	3a (8.1) [−0.6]	1a (1.8) [−2.4]	11a (18.3) [2.0]	6a (13.3) [0.5]	2a (22.2) [1.1]	2a (12.5) [0.2]	2a (11.8) [0.1]
Tobacco status	18	34.99	0.009										
Current tobacco user				5 a (7.5) [−1.7]	2a (3.8) [−1.6]	6b (50) [4.7]	4a, b (10.8) [0.2]	4a (7) [−0.8]	6a (10) [0.0]	4a (8.9) [−0.3]	1a, b (11.1) [0.1]	3a,b (18.8) [1.2]	2a, b (11.8) [0.3]
Past tobacco user				6a (9) [0.3]	5a (9.6) [0.4]	1a (8.3) [0.0]	4a (10.8) [0.6]	2a (3.5) [−1.4]	7a (11.7) [1.1]	1a (2.2) [−1.5]	0a (0.0) [−0.9]	1a (6.3) [−0.3]	3a (17.6) [1.5]
Never tobacco user				56a,b,c (83.6) [1.7]	45a (86.5) [0.9]	5b (41.7) [−3.7]	29a, b,c (78.4) [−0.6]	51a,c (89.5) [1.6]	47a b,c (78.3) [−0.8]	40a, c (88.9) [1.3]	8a,b,c (88.9) [0.5]	12a,b,c (75) [−0.7]	12a (70.6) [−1.3]
Education level	54	37.774	0.95										
Less than high school				0a (0.00) [−0.5]	0a (0.0%) [−4]	0a (0) [−0.7]	1a (2.7) [3.0]	0a(0) [−0.4]	0a(0) [−0.4]	0a (0) [−0.4]	0a (0) [−0.2]	0a (0) [−0.2]	0a (0) [−0.2]
High school				37a (55.2) [1.6]	27a (51.9) [0.9]	7a (58.3) [0.9]	15a (40.5) [−0.7]	25a (43.9)[−0.4]	25a (41.7)[−0.8]	16a (35.6) [−1.5]	4a (44.4) [−0.1]	9a (56.3) [0.8]	7a (41.2) [−0.4]
Post-secondary diploma				5a (7.5) [0.6]	3a (5.8) [0.0]	0a (0) [−0.9]	3a (8.1) [0.6]	5a (8.8) [1.0]	3a (5.0) [−0.3]	1a (2.2) [−1.1]	0a (0) [−0.8]	2a (12.5) [1.1]	0a (0) [−1.1]
Bachelor’s degree				20a (29.9) [−0.7]	14a (26.9) [−1.1]	3a (25.0) [−0.6]	12a (32.4) [−0.1]	19a (33.3) [0.0]	22a (36.7) [0.6]	21a (46.7) [2.0]	2a (22.2) [−0.7]	4a (25.0) [−0.7]	7a (41.2) [0.7]
Master’s degree				4a (6.0) [−1.2]	6a (11.5) [0.4]	1a (8.3) [−0.2]	4a(10.8) [0.2]	5a (8.8) [−0.3]	7a (11.7) [0.5]	5a (11.1) [0.3]	2a (22.2) [1.2]	1a (6.3) [−0.5]	2a (11.8) [0.3]
MD degree				0a (0.0) [−0.5]	1a (1.9) [2.5]	0a (0) [−0.2]	0a(0)[−0.3]	0a (0) [−0.4]	0a (0) [0–0.4]	0a (0) [−0.4]	0a (0) [−0.2]	0a (0) [−0.2]	0a (0) [−0.2]
PhD degree				1a (1.5) [−1.2]	1a (1.9) [−0.8]	1a (8.3) [−0.4]	0a(0) [−0.4]	3a (5.3) [0.5]	3a (5.0) [0.4]	2a (4.4) [0.1]	1a (11.1) [1.1]	0a (0) [−0.8]	1a (5.9) [0.4]

Column percentages presented in parentheses. Policies that differ from one another significantly within each level of each demographic variable at alpha level 0.05 have different alphabets next to the N (Bonferroni correction).

### Factor analysis of the tobacco endgame scale

The item of banning tobacco use for future generations was cross-loaded in two factors. After deleting it, setting the minimum age to 21 was deleted as a redundant item given that setting the minimum age to 25 exists, and deleting the former yielded a unidimensional scale rather than one factor with 7 items and another with 1 item. The final retained factor included eight items (see Table 3). The reliability (Cronbach’s alpha) was very good (*α* = 0.868), a KMO value of 0.909, and the factor (Eigen: 4.3) explained 53.8% of the variance.

**Table 3:** Results for the factor analysis of the Tobacco Endgame Scale.

Item	Factor loading
	1
Factor 1: Support for tobacco endgame policies	
1. Increase tobacco taxes every year by 25%	0.725
2. Standardize cigarette packages such that the entire branding is removed, the package shape is square, and the background color and font is the same on all packages to make them boring and increase attention to health warnings	0.681
3. Initiate lawsuits against the tobacco industry to recover the healthcare costs incurred because of tobacco use	0.696
4. Ban imports of tobacco by 10% every year for 10 years to make tobacco use extinct	0.840
5. Reduce nicotine concentration to the point of reducing addiction	0.731
6. Ban tobacco use for minors and adults	0.793
7. Ban all tobacco flavors	0.802
8. Set the minimum legal age for tobacco use to 25	0.571

The two deleted items were “Set the minimum legal age for tobacco use to 21” and “ban tobacco use for future generations (those who are not currently adults)” while keeping tobacco use legal for current adults.

### Level of tobacco endgame support

The mean tobacco endgame support score was 4.23 (SD: 0.78). In the bi-variable analysis, tobacco use status was the only variable that was statistically associated with tobacco endgame support. Compared to current tobacco users, past tobacco users and never tobacco users exhibited higher tobacco endgame support by 0.55 (BC 95% CI 0.10, 0.97) and 1.00 (BC 95% CI 0.62, 1.38), respectively. Being a female was associated with higher tobacco endgame support by 0.19 as compared males but this was not statistically significant (BC 95% CI − 0.01, 0.40) (Table 4). Similarly, no statistically significant association were observed between tobacco endgame support and age, employment status, and socioeconomic status (Table 4).

**Table 4:** Crude and adjusted associations between Tobacco use status, sex, and other variables and tobacco endgame support level.

	Crude association				Adjusted association			
	β	Bootstrap SE	BC 95% CI	Z, p	β	Bootstrap SE	BC 95% CI	Z, p
Tobacco use status								
Current user	Ref				Ref			
Past user	0.55	0.22	0.10, 0.97	2.49, 0.013	0.89	0.32	0.26, 1.51	2.81, 0.005
Never user	1	0.19	0.62, 1.38	5.19, <0.001	1.33	0.29	0.76, 1.89	4.64, <0.001
Sex								
Male	Ref							
Female	0.19	0.11	-0.01, 0.40	1.77, 0.078	0.57	0.38	-0.22, 1.29	1.50, 0.134
Tobacco use status × sex								
Current user and male	Ref							
Past user and male	0.89	0.32	0.26, 1.49	2.84, 0.005	0.89	0.32	0.26, 1.51	2.81, 0.005
Never user and male	1.33	0.29	0.78, 1.89	4.64, <0.001	1.33	0.29	0.76, 1.89	4.64, <0.001
Current user and female	0.56	0.37	−0.20, 1.28	1.51, 0.132	0.57	0.38	−0.22, 1.29	1.50, 0.134
Past user and female	−0.78	0.43	−1.57, 0.09	−1.81, 0.070	−0.78	0.43	−1.57, 0.08	−1.81, 0.071
Never user and female	−0.66	0.39	−1.38, 0.14	−1.70, 0.088	−0.66	0.39	−1.38, 0.15	−1.71, 0.088
Constant	3.11	0.28	2.58, 3.65	11.05, <0.001	3.11	0.28	2.55, 3.65	11.31, <0.001
Employment status								
Yes	Ref							
No	0.15	0.1	−0.04, 0.37	1.44, 0.149	−0.01	0.11	−0.21, 0.21	−0.03, 0.978
Age (years)	−0.01	0.01	−0.01, 0.01	−0.69, 0.491				
Socioeconomic status								
Low	Ref							
Medium	0.2	0.2	−0.15, 0.63	1.00, 0.317				
High	0.16	0.25	−0.29, 0.66	0.63, 0.526				

Only variables associated with the endgame scores with a *p*-value ≤ 0.20 in crude associations were included in the multivariable analysis. β, observed regression coefficient; SE, standard error; BC, bias-corrected; CI, confidence interval; Z, Z score; *p*, *p*-value; Ref, reference category. Final adjusted model statistics: *n* = 372, bootstrap replications = 3,000, seed number = 12,345, Chi^2^(6) = 45.40, *p* = <0.001, adjusted R-squared = 0.162.

The interaction between tobacco use status and sex was significant. Compared to males who currently use tobacco, males who used tobacco in the past and males that never used it had higher endgame scores by 0.89 (BC 95% CI 0.26, 1.49) and 1.33 (BC 95% CI 0.78, 1.89), respectively. However, there were no significant differences in endgame scores for females who currently use tobacco (0.56; BC 95% CI −0.20, 1.28), females who used it in the past (0.67; BC 95% CI −1.51, 2.86), and females who never used tobacco (1.23; BC 95% CI −0.80, 3.31) compared to males who currently use tobacco.

In multivariable analysis, tobacco use status remained the only variable statistically associated with tobacco endgame support. On average, past tobacco users had higher tobacco endgame support by 0.89 (BC 95% CI 0.26, 1.51) than current tobacco users. Similarly, never tobacco users had a higher tobacco endgame support than current tobacco users by 1.33 (BC 95% CI 0.76, 1.89). No statistically significant and adjusted associations were observed between tobacco endgame support and the remaining variables included in multivariable analysis: sex and employment status. Some interactive terms for tobacco use status x sex were significant—past tobacco using males and males who never used tobacco had higher endgame scores by 0.89 (BC 95% CI 0.26, 1.51) and 1.33 (BC 95% CI 0.76, 1.89) than males who currently use tobacco, respectively. Nonetheless, compared to males who currently use tobacco, no statistically significant interactions in endgame scores were observed among females who currently use tobacco (0.57; BC 95% CI − 0.22, 1.29), females who used tobacco in the past (0.68; BC 95% CI -1.53, 2.88), and females that never used tobacco (1.24; BC 95% CI -0.84, 3.33).

## Discussion

The current study aimed at identifying the associations between sociodemographic variables and preferred choice of endgame strategies and whether or not tobacco use status and sex predict the level of tobacco endgame support in a sample of adult residents in Qatar. The study provides some insights into differences in preferred tobacco endgame strategies by sex and tobacco use status and demonstrates that tobacco use status plays a crucial role in the level of support for tobacco endgame strategies. The preferences for tobacco endgame policies differed across the sample with the top five being tobacco free generations (18%), banning imports of tobacco by 10% for 10 years (15.3%) reducing nicotine to non-addictive levels (16.1%), aggressive reduction of taxes (14%) and banning tobacco use for both adults and minors (12.1%). Past research has demonstrated that reducing nicotine levels was the most supported policy in Kang et al. (28), yet phasing out policies received the highest support levels in Ireland (29). This reveals preferences for different types of tobacco endgame policies varies across countries and warrants further investigation of support levels in other countries. Our study thus contributes new findings that reveal the importance of studying policy preferences in different countries, as those found in our study which was conducted in Qatar differ from those found in other countries. The findings can be explained from the perspective of the HBM. For instance, sex and tobacco use status, both are modifying variables that were related to tobacco endgame policy preferences. Further, tobacco use status and the interaction between tobacco use status and sex both were associated with policy supports. All of these effects depict the importance of modifying variables in determining support levels or preferences for tobacco policies, thereby signifying their role in the context of the HBM. To our knowledge, our study is the first to contribute this novel finding regarding the interactive effect of sex and tobacco use status on tobacco endgame support levels which provides more nuanced information on policy preferences. Below are the results are discussed in detail in relation to the broader literature.

Of all the tested demographic variables, sex and tobacco use status were associated with the preferred choice of tobacco endgame strategies. Specifically, for males, standardized tobacco packaging was the preferred tobacco endgame policy choice relative nicotine reduction policies and vice versa for females. This suggests that males opt for messaging approaches to reducing tobacco use relative to addiction reduction approaches and vice versa for females. The findings add the current literature by going beyond current findings on tobacco control policy perception differences [e.g., (32)] and focuses specifically on tobacco endgame policies. The findings also demonstrate that policy preferences are sex-specific and using multiple strategies may be beneficial to resonate with each sex.

Current tobacco users selected standardized tobacco packaging as their preferred tobacco endgame policy relative to most of the other tobacco endgame policies. This is a logical finding given that current tobacco users are exposed to cigarette packages than never tobacco users and value its benefits. Given the literature on the effectiveness of plain packaging in reducing tobacco use Al-Hamdani (12), this finding is promising as it suggests that tobacco users in Qatar welcome standardized packaging rendering it a good option for the country.

Never tobacco users preferred progressive reduction in tobacco product imports by 10% for 10 years, full-on ban on tobacco use for both minors and adults, and the imposition of a yearly 25% tax on tobacco relative to standardizing tobacco packaging. The progressive reduction of tobacco imports with the final goal of eliminating imports and full-on ban on tobacco are eliminator approaches and may stem from the distaste of never tobacco users for tobacco products and presents strong willingness to eradicate tobacco use. The preference for high yearly tobacco tax increases is also an aggressive policy measure and the implementation of a 25% increase in taxes followed by annual increases by 12.5% is associated with reductions in tobacco use such as Australia (4). Given that never tobacco users constitute the largest segment of the population in Qatar, this sample of participants suggest that most people will be in favor of innovative and aggressive tobacco endgame approaches in the country.

We assessed tobacco endgame policy support through a newly introduced scale, which demonstrates good reliability and structural validity. This tool can be further tested with larger and more diverse populations in the future. For example, the support for tobacco endgame policies can be compared between countries and evaluated in light of existing tobacco control policies and cultural norms that oppose tobacco use. In this study, we found that never tobacco users have higher levels of support for tobacco endgame strategies relative to past and current tobacco users. This finding is consistent with findings from the broader literature (25, 26), yet shows that the same level of support extends to tobacco endgame policies, which unlike conventional tobacco control policies have a goal for reducing tobacco use to near negligible levels. As mentioned above, given that most of the public are never tobacco users, it is likely that tobacco endgame policies will be well endorsed by the public. This finding like the ones above that pertain to never tobacco users are promising indicators of tobacco endgame support in most of the population. Further, males who do not currently use tobacco (both never users and past users of tobacco) report higher support for tobacco endgame strategies, which suggests that they are more likely to comply with tobacco endgame strategies relative to males who currently use tobacco. Supports for quitting tobacco use, such as cessation support, may therefore be necessarily to support males who currently use tobacco for effective tobacco endgame implementations.

A number of limitations exist. First, tobacco use is a sensitive topic to speak about in a university setting which limited the collection of specific data such as faculty vs. students. This limited nuanced analysis by such demographic variables. Second, we did not include some tobacco endgame policies, which need to be added and tested in future studies that examine preferences for tobacco endgame strategies. Future studies need to modify the scale based on a thorough examination of a wide range of tobacco endgame policies to make the scale more inclusive. Specifically, future investigations need to employ more comprehensive approach for content validity including input from experts in tobacco endgame policies, a thorough review of tobacco endgame policies listed by major organizations, for example: the WHO (36, 37). Further, a Confirmatory Factor Analysis (CFA) should be conducted with a separate sample to confirm the factor structure for construct validity. Third, the sample we used was not tested at the population level but rather a convenience sample; the results cannot be generalized to the population.

## Conclusion

Tobacco-free generations was the top preferred strategy across the sample in this study, which was comprised of adults who reside in Qatar. This demonstrates that preferences for endgame strategy type is based on country of residence as past studies identified other strategies that were preferred by adults. Males prefer tobacco endgame strategies that focus on reminders for health harms in tobacco packages while females prefer strategies aimed at reducing nicotine levels in an effort to reduce addiction thereby calling for diverse strategies to reach different sexes. Never tobacco users report a preferences for a wide range of tobacco endgame policies relative to those with past tobacco use history in addition to reporting higher levels of support overall which suggests that social acceptability for the tobacco endgame in Qatar is high. Further, males who do not currently use tobacco report higher support levels than males who currently use tobacco. Therefore, there is a need for cessation supports along with tobacco endgame implementation to support males who currently use tobacco.

## Data Availability

The datasets presented in this article are not readily available because the data that has been used is confidential. Requests to access the datasets should be directed to Mohammed Al-Hamdani, malhamdan@qu.edu.qa.
